# “You Just Forge Ahead”: The Continuing Challenges of Disaster Preparedness and Response in Long-Term Care

**DOI:** 10.1093/geroni/igab038

**Published:** 2021-09-18

**Authors:** Lindsay J Peterson, Debra Dobbs, Joseph June, David M Dosa, Kathryn Hyer

**Affiliations:** 1 Florida Policy Exchange Center of Aging, School of Aging Studies, University of South Florida, Tampa, Florida, USA; 2 Center of Innovation in Long-Term Services and Supports, Providence Veterans Affairs Medical Center, Providence, Rhode Island, USA; 3 School of Public Health, Brown University, Providence, Rhode Island, USA

**Keywords:** Assisted living, Disaster preparedness, Nursing home, Qualitative

## Abstract

**Background and Objectives:**

Protecting nursing home and assisted living community residents during disasters continues to be a challenge. The present study explores the experiences of long-term care facilities in Florida that were exposed to Hurricane Irma in 2017.

**Research Design and Methods:**

We used an abductive approach, combining induction and deduction. Interviews and focus groups beginning in May 2018 were conducted by telephone and in person with 89 administrative staff members representing 100 facilities (30 nursing homes and 70 assisted living communities). Analyses identified themes and subthemes. Findings were further analyzed using the social ecological model to better understand the preparedness and response of nursing homes and assisted living communities to Hurricane Irma.

**Results:**

3 main themes were identified including: (1) importance of collaborative relationships in anticipating needs and planning to shelter in place or evacuate; (2) efforts required to maintain safety and stability during an unprecedented event; and (3) effects, repercussions, and recommendations for change following the disaster.

**Discussion and Implications:**

Preparing for and managing disasters in nursing homes and assisted living communities involves actions within multiple environments beyond the residents and facilities where they live. Among these, community-level relationships are critical.

**Translational Significance:** Long-term care facilities continue to face challenges in protecting residents in disasters. Adequate preparedness and response requires action on multiple levels, ranging from within the facility to public policy. Connections with stakeholders at the community level are critical, and there is a particular need for more effective collaboration between long-term care and community emergency preparedness agencies.

Research has documented the dangers of severe weather to residents in long-term care. Studies have found that nursing home (NH) residents exposed to one of four hurricanes that struck the United States in 2005–2008 were more likely to die or be hospitalized than residents in nonexposed comparison groups (Dosa et al., 2012) and that mortality and morbidity were higher among residents who were evacuated (Castle & Engberg, 2011; Willoughby et al., 2017). Recent research found higher 30- and 90-day mortality and hospitalization among NH residents who experienced Hurricane Irma in 2017 compared to residents from prior years without storm exposure (Dosa et al., 2020). In a similar analysis involving assisted living communities (ALCs), residents exposed to Hurricane Irma had higher rates of emergency department use at 30 and 90 days after the storm (Hua et al., 2021). These studies suggest there continues to be a need for knowledge of how to protect NH and ALC residents whose health and safety are at risk in a hurricane or other potential disaster.

Hurricane Irma measured 400 miles across before landfall. After crossing the Florida Keys, it made landfall in southwest Florida as a Category 3 storm with a wind field that threatened the entire state as it travelled north (Cangialosi et al., 2018). Among those at risk at the time were an estimated 71,000 residents of Florida’s 670 NHs and 85,000 residents of the 3,112 ALCs (Jester et al., 2021; Peterson et al., 2020). In the present study, we conducted qualitative interviews and focus groups with owners and administrators of NHs and ALCs in Florida concerning their experiences related to Hurricane Irma, including actions they took to prepare for the hurricane and challenges they faced during and after the storm.

The social ecological model (SEM) guided this study (Figure 1). As a model, SEM views individuals as nested within larger systems or environments that influence their development and outcomes (Sallis et al., 2008). This model is often presented as concentric circles, with the individual at the center and the influences on that individual (e.g., neighborhood, society) depicted by circles radiating outward. It has the potential to provide a more holistic view to show how actions in one environment depend on, or may influence, actions in another (Bronfenbrenner, 1986).

**Figure 1.**
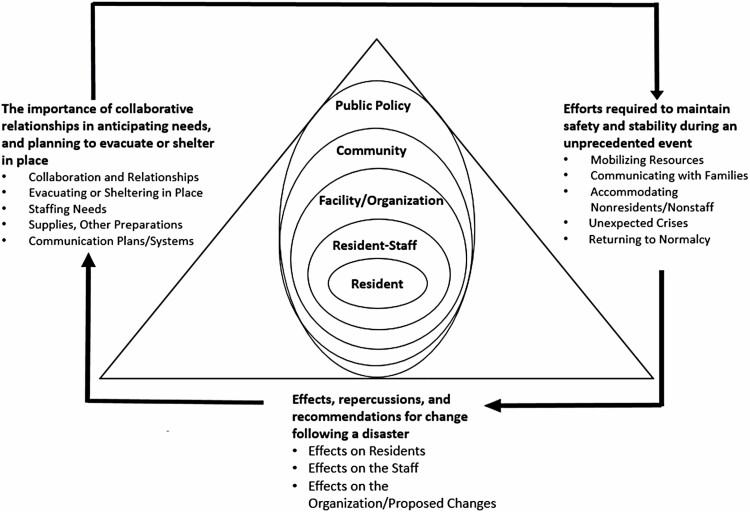
Social ecological model of disaster preparedness and response in long-term care.

The SEM has been adapted to study disaster vulnerability (Thomas et al., 2013) and further, to study home-based care provided to older and disabled adults after the 2017 Hurricane Maria (Haverhals et al., 2020) and long-term care services for veterans (Haverhals, 2016). Bell and colleagues (2021) used an adaptation of the SEM to study home-based care provided to older adults after Hurricanes Harvey, in 2017, and Irma. The present research applied the SEM to an examination of the factors that may have influenced preparation for and response to Hurricane Irma in NHs and ALCs. Our model included the social, physical, and organizational aspects of the long-term care environment, in addition to the larger community and still larger public policy environments in which long-term care facilities operate.

## Protecting Residents of NHs and ALCs in Disasters

Studies of Hurricanes Katrina (2005) and Gustav (2008) and hurricanes that struck Florida in 2004 described the multiple challenges of keeping NH residents safe during a disaster, including maintaining staffing, supplies and power generation, and evacuation safety (Dosa et al., 2008; Hyer et al., 2009; Laditka et al., 2008). Evidence from other studies suggests NHs have not been regarded as health care settings and not engaged in community disaster planning or collaboration with emergency management agencies (Laditka et al., 2008; Pierce et al., 2017; Rhoads & Clayman, 2008).

ALCs also warrant attention, given this population’s acuity (Harris-Kojetin et al., 2019). Brown and colleagues (2015) surveyed Florida ALC staff exposed to hurricanes in 2004–2005, reporting their challenges were similar to those of NHs. However, other research identified differences between the two settings, with a study in Texas after Hurricanes Katrina and Rita (2005) finding ALCs were more likely to report difficulties obtaining needed supplies and equipment (Castro et al., 2008). An Ohio study based on a statewide survey found ALCs to be less prepared for disasters than NHs (Kennedy et al., 2021). Indeed, NH and ALC regulations differ overall, with ALCs regulated by the states and subject to requirements that may be less prescriptive than federal NH regulations (Hyer et al., 2007).

Research of the last decade led to policy recommendations emphasizing multiple factors that are critical to disaster preparedness in long-term care (Florida Health Care Association, 2007). The purpose of this study was to use the SEM to better understand preparedness for and response to Hurricane Irma in NHs and ALCs from the perspective of administrative staff and owners. This approach has the potential to provide knowledge of the factors to be taken into account across multiple environments in any effort to improve the safety and well-being of long-term care residents exposed to disasters.

## Method

### Sample

A convenience sample of Florida NH and ALC administrative staff and owners was recruited to participate in this study. Our research team attended conferences and workshops of statewide provider membership groups (e.g., the Florida Health Care Association, Florida Assisted Living Association), obtaining names of those interested and contacting them by electronic mail correspondence. We employed maximum variation techniques (Palinkas et al., 2015). In keeping with this method, which aims to ensure inclusion of varied stakeholders, we sought participants from NHs and ALCs that differed by evacuation status (sheltered in place or evacuated) and region within Florida. Additionally, we recruited ALCs by size (<25 beds and 25+ beds), based on previous research indicating differences in practices based on size (Caffrey et al., 2014).

### Data Collection

Data were collected through individual interviews and focus groups by telephone and in person. We used both because we sought information from varied perspectives and not all participants were available for focus groups. With each interview and focus group, we used one interview guide including questions about the decision to evacuate or shelter in place, collaboration with emergency management and other government officials, assistance sought or received, resident experiences, and power loss experiences (see interview guide in Supplementary Material).

We conducted 61 telephone interviews and six in-person focus groups of three to seven individuals each (*N* = 28) for a total participant sample of 89. Data were collected between May, 2018 and January, 2020, with a majority of the interviews and focus groups taking place in the first 7 months but data collection continuing to ensure the inclusion of facilities from diverse geographical areas and facility types. NH participants were combined with other NH participants in focus groups, as were ALC participants, in all groups except for one in which NH and ALC participants were combined. Some participants (e.g., owners, regional administrators) discussed experiences at more than one facility. Together the participants represented 100 facilities (30 NHs and 70 ALCs). The individual interviews took 40–50 min, and focus groups took 60–90 min each and included a gerontologist or sociologist PhD-trained facilitator and note-taker with expertise in disasters and long-term care research. Facilitators and note takers discussed field notes after each group. Five of the focus groups were conducted in English and one ALC group was conducted in Spanish by a team member who was fluent in English and Spanish. Each interview or focus group participant received a $35 gift certificate. All interviews and focus groups were audio-recorded and professionally transcribed verbatim. The Spanish language focus group was professionally transcribed in Spanish, and then translated to English. All transcripts were entered into Atlas.ti 8.0, a software product used to manage and analyze qualitative information. The protocol and study materials were approved by the University of South Florida Institutional Review Board.

### Analysis

In this study, we use an abductive analytic approach (Bell et al., 2021), a method for analyzing data that combines inductive and deductive approaches allowing for the purposeful examination of a range of explanations, thereby reducing the likelihood of bias. The abductive approach allows for a creative inferential process aimed at producing new models based on study results (Timmermans & Tavory, 2012). We used the COnsolidated criteria for REporting Qualitative research checklist for reporting of qualitative research to ensure methodological rigor (Tong et al., 2007). These methods included the use of a theoretical framework, audio-recording and note-taking of interviews and focus groups, a team-coding approach, memoing to document theme development, regular reconciling team meetings, and member-checking of themes.

Four members of the research team (L. J. Peterson, D. Dobbs, J. June, and D. M. Dosa) trained in sociology, gerontology, geriatrics, public health, and communication jointly coded the first 10 interviews and two focus groups to establish a set of codes. Once an initial code list was generated, a two-person team coded each interview and focus group. A five-member research team provided input on discrepant codes until a clear and agreed-upon description of codes resulted. Research team members met weekly to discuss codes and resolve discrepancies, revising the list, updating code definitions, and adding new codes or consolidating related codes as main themes and subthemes were identified. Analytic decisions and steps, such as consolidating codes and identifying themes, were documented throughout the process. For the member-checking, we distributed theme descriptions and exemplar quotes to eight study participants and our advisory board committee of state and national experts, who were assembled to guide a larger disaster preparedness research project at the university. Based on feedback, revisions were made to clarify our definition of collaboration.

In the final analytical step, after theme and subtheme identification, we analyzed these results in the context of our conceptual model. This was done to better understand the relationships of our themes and subthemes to the varied environments (e.g., organizational, community, public policy) potentially affecting the experiences of long-term care facilities and their residents exposed to Hurricane Irma.

## Results

Characteristics of the NH and ALC participants (*N* = 89) are reported in Supplementary Table 1. Over half were female and identified as White, non-Hispanic. Most were administrators; nearly one-quarter were owner/administrators (from ALCs). Forty percent had 6–20 years of experience in long-term care, and 60% had prior hurricane experience. The facility characteristics are given in Supplementary Table 2. Half of the sample evacuated for Hurricane Irma. The 32 small ALCs had an average of nine beds, and the 38 large ALCs had an average of 113 beds each. About 44% of small ALCs accepted Medicaid (no data were available on percent of residents receiving Medicaid and ALC occupancy rates), 41% were part of a corporate chain, and all were for-profit. Of large ALCs, 53% accepted Medicaid, 58% were part of a chain, and 74% were for profit. About 15% of small ALCs offered memory care; one-third of large ALCs did so. Of the 29 NHs, the average bed size was 128 with 88% occupancy. Nearly 52% were for-profit, 73% were chain-owned, and 35% had beds for residents with dementia. Medicaid occupancy averaged 52%.

We identified three main themes from the data, organized around the time points of Hurricane Irma: (1) importance of collaborative relationships in anticipating needs and planning to shelter in place or evacuate; (2) efforts required to maintain safety and stability during an unprecedented event; and (3) effects, repercussions, and recommendations for change following the disaster. Table 1 provides quotes illustrating themes and subthemes.

**Table 1.** Themes, Subthemes, and Hurricane Irma Experiences of Nursing Homes and Assisted Living Communities

Subtheme	Assisted living	Nursing home
Theme 1: Importance of collaborative relationships in anticipating needs and planning to shelter in place or evacuate		
Collaboration and relationships	*Small, Evacuated*: I think the closest partnership that I have would be from relocating from one of my facilities to another [Iown]. *Small, Sheltered in Place*: Every year we have to send an emergency plan to the [county], but they never appeared to try to help. *Large, Sheltered in Place*: I joined the [County] disaster preparedness group. ... I think maybe there was only one other assisted living center that was a part of that.	*Evacuated*: We did work closely with [the local EOC director] ... He was very responsive. *Sheltered in Place*: We’re really fortunate that the EOC office in our area is very easy to work with, and have table-top exercises every year. *Sheltered in Place*: We have an emergency command center at our corporate office.
Decision to evacuate or shelter in place	*Small, Evacuated*: We always evacuate ... because of the power outages and the unpredictability of the staff. *Small, Sheltered in Place*: I’ve stayed in [my] house for many hurricanes over the years so I knew the house would be fine. *Large, Evacuated*: We’re right in the zone for mandatory evacuations. The liability would be too high if something would happen. *Large, Sheltered in Place*: I was not inclined to want to take a facility of 65 people and all of our meds and staff and food and drive through the middle of the state to [evacuate]. We’re in the last [zone].	*Evacuated*: I go by what’s in our plan. Our plan is that we evacuate at Level A. *Evacuated*: There were a number of storms where I refused to evacuate, and it was always the right call. But these days, they pretty much, you know, if you don’t and one resident gets a scratch, you’re going to be on the news and persecuted. *Sheltered in Place*: It’s really disruptive to have to evacuate the residents. Very stressful on them, on the staff. ... It’s labor-intensive, it’s financially challenging. There are so many logistical implications to it as well. So if you can shelter in place safely, that’s the best thing to do.
Staffing needs	*Small, Sheltered in Place*: I tried to have all my employees at the place during the storm because in case we have to do something I want to have all the employees working. *Large, Evacuated*: Weeks before, we had set up a lot of different systems for who was going to shelter in place or stay with the community, and who was going to go ... We created all these spreadsheets for all this kind of stuff.	*Sheltered in Place*: Prior to a hurricane, understand who’s on your A team and who’s on your B team. ... Try to narrow it down to these are the folks that we really want here during the hurrican. ... Understand all of those dynamics before putting your schedule together. *Evacuated*: [Staff] already know we’re on an island. [I tell them] at hurricane 2 level we pack up all the residents and we go and we stay usually about five days, and I tell them it’s hard work.
Supplies, other preparations	*Small, Evacuated*: We make a pre-packed bag [of clothes and toiletries] and we want to keep it in the closet ... I actually do it because of being a mom. *Small, Sheltered in Place*: [We had] hurricane panels, we had generator, water and all the stuff that we are supposed to have. So, basically we are always ready for the hurricane season. *Large, Sheltered in place*: We would always prepare every year starting in June ... making sure all the facilities were fully prepared	*Evacuated*: We meet once a month for at least an hour a day and our agenda is pretty full. We have checklists, long checklist, of food and medical supplies, personal care supplies, water, hotel, transportation, personnel, A and B team. *Sheltered*: 100% of all of our buildings were on generator power. *Sheltered in Place*: Have an automatic delivery, once there’s a warning or even prior to that. They deliver extra wipes, extra nursing supplies, so that you don’t run out.
Communication plans/systems	*Small, Evacuated*: I made sure I had everybody’s information in my phone, and the number that they wanted to be contacted on, via text and phone call, because landlines can go down, cell lines can go down. *Large, Evacuated*: We have (an) emergency communication disaster system. It gets plugged in when something happens.	*Evacuated*: Our social worker actually called all family members to give them the address, location, phone numbers where they would be able to contact staff. Our staffing phone is monitored 24 hours/day, we gave that phone number. My cell phone number was given out as well. *Sheltered in Place*: Our corporate office was ready to update [families] in case we lost power and couldn’t get on Facebook, because a lot of our families know that we have a Facebook page and everything.
Theme 2: Efforts required to maintain safety and stability during an unprecedented event		
Mobilizing resources	*Small, Evacuated*: It was really myself, my daughter, and [a lady who helps me clean] that carted all the stuff up in the elevator and across the building to where we were staying. *Small, Sheltered in Place*: Setting up beds, boarding up the house. I mean, I could go on forever. There’s a lot of work that goes into preparing a house. ... There’s just a plethora of information, of tasks that we do. *Large, Evacuated*: We were so lucky in that we got more staff than we needed. I mean they were amazing. *Large, Sheltered in Place*: I had to be right in there with them doing everything between cleaning toilets and giving showers ... I was just one of them at that time.	*Evacuated*: We had our COO down there, who is a nurse. He is a nurse, was down there helping us start IV. ... We had a corporate attorney down in our building. Our Vice President, that was down there. Every hand was in the pot. Everyone was helping out. *Sheltered in Place*: We did have some people on the A list leave. And we had some people on the B list decide to come in to shelter in place. And that was part of what we were communicating to them. “You can bring your family, you can bring your pets ... But if you come, you’re working, we can’t have you in the building not working.” *Sheltered in Place*: There was no assistance [from the state or emergency management].
Communicating with families	*Large, Evacuated*: In the beginning, certainly they [were] still able to call our community, but then once we vacated and we closed the community, all phones were then rerouted to corporate. *Large, Sheltered in Place*: We were communicating with everyone via the land line. Once we lost all power to the phones, we then had a community cell phone.	*Evacuated*: I thought we had a good plan, and part of that was going to be on social media. [The company] was going to put out information to folks if we weren’t able to communicate. [But] people just didn’t know …where to look. It wasn’t as efficient as I thought it might be.
Accommodating nonresidents, nonstaff	*Small, Evacuated*: There were at least 12 family members ... from about 12 months old baby up to teenagers. *Large, Evacuated*: The neediest were the uninvited guests, which was another 50 people. The first thing they wanted was the WIFI. Second thing they wanted was what’s for dinner. *Large, Sheltered in Place*: We had more than what we needed or wanted in the way of family members [of staff]. But at the time, we were not in a position to turn people away. So we had great hands on deck for care, but ... more family in the building than what we had planned in terms of food.	*Evacuated*: We had families that showed up at the door [at the host site], that, even though we was at max capacity, we was just letting them in. That was the safest place for them at the time because we had the generator. We had food. We had water. *Sheltered in Place*: The spouses could come in, but they had to bring their own stuff. We would feed them the day before, the day of and the day after the hurricane, but after that they were on their own. *Sheltered in Place*: We actually brought in sod. We put down plastic in the stairwells on the ground floor and put sod in for [pet] potty breaks during the storm. Because, they didn’t want us opening up doors.
Unexpected crises	*Small, Sheltered in Place*: In my case, I panicked. They were painting a hurricane that was going to destroy us. But there was nowhere to go. *Large, Evacuated*: When push came to shove, one of the communities would not accept any of my residents ... They didn’t have any room. *Large, Sheltered in Place*: I had two insulin-dependent residents, who home care was coming doing Accu-Cheks and administering insulin. [After news broke that the storm was tracking toward the ALC] and they said, “We’re not coming.”	*Evacuated*: We were telling [the EOC official] about the challenges of evacuating, he kept telling us to refer to our evacuation plan. And we told him, “Our evacuation plan does not cover when the governor evacuates the entire state with no fuel.” ... And we asked him, would he sell us some fuel ... He said, “No, you’re on your own, and make sure you leave.” *Sheltered in Place*: I literally worked the floor with 60 residents by myself [as the only nurse] for 49 hours.
Returning to normalcy	*Small, Evacuated*: We stayed a week after the storm because my facility did not have any electricity. So I wasn’t going to move them from one facility with no power to another facility with no power. *Small, Sheltered in Place*: We need to make sure that we get those shutters off as quickly as possible [to let in the light]. *Large, Evacuated*: [Residents were] very grateful to be back in their own home and bed. I think there was a bit of a shock after having gone through everything. Folks didn’t start coming out of their apartments and start resuming normal life for probably another couple of days after we returned.	*Evacuated*: What we did was, after about four or five days, we start moving a little bit at a time back to [NH]. At that time we was sending about five or six [residents] a day, until we got back fully staffed, because a lot of staff left. *Evacuated*: We had to wash everything else, which took 24 hours a day for three days, to wash all the linen and everything. ... Getting them their correct mattress and their correct wheelchair, that was stressful to them, because they like their familiar objects. That took a week to iron all that out.
Theme 3: Effects, repercussions, and recommendations for change following a disaster		
Effects on residents	*Small, Evacuated*: One [dementia] resident went to a nursing home. She left walking and talking. ... She came back in a wheelchair. *Large, Sheltered in Place*: [During the storm] one ran out to go and got into his car ... and I was behind him and it was pouring rain. [He was] aggressive, because he was not in his environment.	*Evacuated*: I think it had a terrible effect. We did not have anybody drop dead then, but there were people who never quite came back [mentally] ... People who were far away when we brought them there, moved farther away from reality. *Sheltered in Place*: At 6 am when it became evident that the storm surge had come and gone and we were safe, we took them back [down to the first floor]. And they did not sleep well. They were extremely confused.
Effects on staff	*Small, Sheltered in Place*: I had more issues with staff and staff’s children being terrified. *Large, Evacuated*: I must have lifted 150 people. *Large, Sheltered in Place*: I’m a much older woman, carrying people and moving mattresses and staying up all night for three days ... as soon as I would fall asleep, somebody would wake me.	*Evacuated*: [One CNA] the epitome of your just, you know, that shining star ... As the storm was getting closer, she started to panic. *Sheltered in Place*: [After the hurricane] we got five surveyors walked in to do the annual survey. ... None of us could recuperate from that seven, eight, nine days of hell. *Sheltered in Place*: It was scary. I was just really scared, I just didn’t expect it to hit there and tear the roof off and the side.
Effects on organization/proposed changes	*Small, Evacuated*: I would do more activities to relieve the sense of, you know, being upset and not in their homes ... distract them. *Large, Evacuated*: There needs to be more assessment [of the buildings] to say, “Yeah, this building is 20 years old, it’s not safe. This building is three years old and these criteria are met.” *Large, Sheltered in Place*: They should have a liaison at our emergency management office, just for assisted living.	*Evacuated*: We should have a right to go to those shelters, because our emergency evacuation plan calls for us to go to buildings all over the state. In a ... mass evacuation, that’s impossible. *Sheltered in Place*: I think the nursing board needs to be a little bit more stringent, or give different sanctions on nurses who decide not to show up. CNAs and all that, that’s a different story. But licensed nurses ... should be a role model.

*Note*: ALC = assisted living community; COO = chief operating officer; CNA = certified nurse assistant; EOC = emergency operations center; IV = intravenous line; NH = nursing home.

### Theme 1: Importance of Collaborative Relationships in Anticipating Needs and Planning to Shelter in Place or Evacuate

All NH and ALC participants had prepared to some extent for Hurricane Irma. We identified five subthemes showing how they varied in their preparedness efforts. The subthemes are as follows.

#### Collaboration and relationships

Several participants discussed relationships or partnerships created as part of disaster planning, including working with local emergency officials and/or regional health care coalitions that conducted regular disaster planning discussions. NH administrators were more likely to mention these relationships than ALC administrators. One ALC participant said she received walkie-talkies from local emergency management officials. However, few ALC participants noted any contact with emergency management officials beyond submitting their annual disaster plans. Many NH and ALC participants emphasized their dependence on corporate and private relationships, exemplified by an ALC administrator’s comment that he was “lucky” to have “corporate structure and support” to help him with disaster preparation.

#### Evacuating or sheltering in place

State-required disaster planning includes considering whether to evacuate or shelter in place. Most participants said they planned to evacuate if ordered to do so because of their location. Evacuation orders typically apply to coastal areas subject to flooding in severe weather. However, many of the NH and large ALC participants said they sought to shelter in place if possible. Said the administrator of a large coastal ALC: “We will shelter in place because of the post-traumatic stress for residents, the volatile residents.” One of the NH administrators discussed planning to shelter in place because he was in an area that has never required evacuation. However, others who also were outside of evacuation zones chose to evacuate because they were unsure of their building safety, as this administrator said: “Our building wasn’t a year old. There was several issues with our roof, from leaking.”

#### Staffing needs

Long-term care facilities in Florida are directed to develop disaster staffing plans (Florida Agency for Health Care Administration, 2021). However, plans varied in the extent to which administrators appeared to think through their staffing needs. Some established specific procedures, as exemplified by the following from a large ALC that sheltered in place:

We had our A teams and B teams. [Managers] completed documents to say at the beginning of hurricane season that we are essential employees [expected to] work throughout the hurricane and bring your family.

Similarly, others emphasized they informed staff of their expectations, as in the case of this NH administrator: “When I hire somebody, I tell them that we evacuate for hurricanes.” Among the small ALCs, only one mentioned formal prestorm staffing plans. Some of those who owned two or three communities planned to consolidate staff and residents in one location, even if it meant evacuating from areas not under evacuation orders. As one participant stated, she thought it would be easier to allocate responsibilities with all staff together. “We had the option of keeping them separate in different homes, (but) I could not be in charge of them if I was not able to be in the same location.”

#### Supplies, other preparations

Some of the NHs and larger ALCs described extensive precautions that enabled them to more securely shelter in place (e.g., supply stockpiles, hurricane windows, power generators). Noted one participant from a large ALC, “Our critical switch gear and electronics are on the second floor, so we could ... sustain a loss of the first floor.”

In comparison, the preparations for many of the small ALCs were more basic, dealing mainly with obtaining extra food and water, as described by this administrator who sheltered in place but had limited ability to make structural changes: “[The state] requires that you have to have emergency food and water for 3 days. I always have plenty more than 3 days. My concern would be that it would blow the house [away].” However, some small ALCs were thorough in their anticipation of residents’ needs, exemplified by this comment: “You have to have cat litter ... You kind of place a Walmart bag in toilet, put kitty litter in them and people can use the toilet and then you can just throw that away.”

#### Communication plans/systems

Most NHs and ALCs established communication plans, but as with other plans some were more extensive than others. One large ALC, for example, planned to produce social media videos to demonstrate residents were safe during the hurricane. Some participants discussed relying on corporate offices unaffected by the hurricane to update Facebook pages. Many others, however, small and large, depended on landline and cellular telephones.

### Theme 2: Efforts Required to Maintain Safety and Stability During an Unprecedented Event

All NH and ALC participants described the events immediately before and after Hurricane Irma. Their comments revealed varying abilities to maintain safety and stability as they confronted the emerging challenges, as differentiated in five subthemes.

#### Mobilizing resources

A majority of participants from NHs and ALCs, both small and large, discussed the challenge of mobilizing supplies and other resources as the hurricane approached. Staffing was the most prominent concern, as exemplified by this comment from the administrator of a small ALC that evacuated:

One of the hardest things was putting the staff at ease that they need to come to work, they need to be responsible that way, bring their families if they have to. A lot of them thought well, they got the order to leave town.

Those who evacuated seemed to have more difficulty maintaining staffing than those who sheltered in place and could provide staff with familiar accommodations. Said one administrator of a NH that evacuated:

You can [require people to work] but the reality is, there’s someone who’s not going to ... We just went ahead and collected names and said, “Look, if you’re willing to come with us ... we’ll go ahead and keep you on the clock through this process.”

#### Communicating with families

During and after the hurricane, many NHs and ALCs had difficulties communicating with family members, as exemplified by this administrator of a small ALC that sheltered in place: “We were trying to do either voice mail or text. They weren’t coming in immediately. ... Sometimes they might not get them for days.”

Larger facilities also had difficulties, including this NH that sheltered in place, “There was a period of time where we didn’t have a landline,” said the administrator. “After the storm, our social worker was reaching out to people a lot of times by e-mail, because, again, phone service was sketchy.”

#### Accommodating nonresidents, nonstaff

To persuade employees to work during the hurricane, nearly all participants offered shelter to staff members’ families, including pets. However, managing visitors posed challenges. One administrator of a large ALC described his experience with “a group that came in [from another facility] that said they were a staff’s family member that actually wasn’t. ... They were doing drugs in one of our stairwells.”

Others, however, managed without incident, such as this NH administrator whose facility sheltered in place, “It was like 203, 204 [people] in the building, and then there was 16 animals. ... iguana. A couple hamsters. Except for [dogs] barking a couple times, honestly, there was no issue.” Some discussed their determination to maintain order, such as this NH administrator. “I said, [to family members] You’re here to work, you’re not here to just have a free stay, you’re gonna pass water ... you’re gonna whatever.”

#### Unexpected crises

Administrators of NHs and ALCs across the state faced multiple crises because of the size, strength, and unpredictability of Hurricane Irma. Many described losing access to planned evacuation destinations, facing last-minute evacuation orders, and/or lacking electrical power for extended periods. As a participant from a small ALC described, “Our Plan B wasn’t working because we could not go to another facility. You had to have Plan C. That was going to a [public] shelter.”

Several described turning to friends and neighbors when help from public agencies was unavailable. A participant from a NH that was ordered unexpectedly to evacuate described using Facebook to find help moving residents onto buses.

A bunch of guys ... showed up and lifted residents, because only one of the buses has a wheelchair accessible ramp, so we had to carry the rest of them up the stairs because that’s all we had available.

A NH administrator who sheltered in place called vendors and others for help when one of his generators failed after the storm. “We said, ‘We need ice. We need fans ....’ [Soon] three or four vans pulled up. It was a lot of the staff, and they all came with their kids.”

NH and ALC administrators who were well connected through their corporations or prior experience appeared to be better prepared to respond. As one ALC administrator who needed ice explained, “We went to the ice company….We knew what we had to do.”

#### Returning to normalcy

In the days after Hurricane Irma, NHs and ALCs faced additional challenges to reestablish normal operations. Extended power losses delayed this process for many, including this administrator of a small ALC.

[I] drove around and whenever I saw an [electric company] truck, I would beg them, “Please, speak to your supervisor. We are an assisted living facility ... we have people that require oxygen or a nebulizer ... It was falling on deaf ears.”

Others that evacuated were delayed in returning to their NHs or ALCs because of confusion about when they could return, as exemplified by this comment from the administrator of a large ALC who needed approval from the local fire marshals: “That wasn’t something that they were willing to do ... There was no real assistance.”

One NH administrator whose facility evacuated described her own efforts to help the host facility return to normal:

We did what we could to help, and that goes for when we left. I made sure everything was cleaned up as it was when we got there, even better. We don’t want to be the ones that are not asked back.

### Theme 3: Effects, Repercussions, and Recommendations for Change Following the Disaster

The disruption and damage of Hurricane Irma affected residents and staff members of many NHs and ALCs. The experience also highlighted planning gaps that led to operational and organizational changes and recommendations for change.

#### Effects on residents

Many participants discussed the anxiety some residents experienced, especially residents with dementia whose routines were disrupted when they were evacuated. “We had a lot of psychiatric episodes,” said the administrator of a large ALC that evacuated. Another ALC administrator who evacuated described the need to tranquilize residents, saying “this is very emotional, because older people, they don’t know if they’re going to see next day.” The physical difficulties of evacuating were evident in this comment from a NH administrator. “We had 90–100 residents that were in frail condition lying on mats [in a shelter for days].” Another administrator described residents returning home after an evacuation in a bus with no air conditioning. “It was not pleasant.”

By contrast, many who sheltered in place described distinctly different experiences, aided by their efforts to distract and entertain residents while they sheltered. As one administrator of a large ALC said, “We had people playing music and singing, and we had the patio furniture in the lobby. It was fun.” However, sheltering in place also posed risks, as described by an ALC administrator with a resident at risk of heat stroke when power was lost.

We ... put her in a bed, wet towels, pushed fluid on her because she wasn’t drinking, put a cooler on her actually. We got her an individual cooling unit and put it next to her in her room and helped her through that little crisis.

#### Effects on staff

Staff members also experienced anxiety, as exemplified by this comment from an administrator of a small ALC that evacuated: “I was crying for months [afterward]. I was so mentally exhausted, I was like literally in pieces for months.” The administrator of a large ALC that sheltered in place discussed the challenge of keeping her staff on an even keel. “You just remain calm, communicative, and then you just forge ahead.”

#### Effects on the organization/proposed changes to disaster preparations

The most widespread change to emerge from Hurricane Irma was a state rule requiring all NHs and ALCs to have alternate power sources (e.g., generators) to operate air cooling systems during power outages (Reedy, 2018). The new law was difficult for many to implement, as one NH administrator said: “The bureaucracy you have to go through to get a generator approved ... is ridiculous.” However, many conceded the new law will improve future preparedness: “I agree with the heart of the rule and the thought process behind it,” said an administrator of a large ALC.

Participants discussed other logistical and operational changes. Prominent among them were measures to improve staffing and attend to staff members’ needs during a hurricane, as exemplified by the following from an administrator of a large ALC that evacuated:

As well as I think it went, we have implemented mandatory rest periods, mandatory meetings, dining times, again to protect the welfare and well-being of our staff that would work nonstop, 24 hours after 24 hours.

Others called for a change in policies that require long-term care facilities to evacuate to similar facilities, stating that suitable destinations often require hours-long drives or become unavailable in a storm with widespread effects. As exemplified by the following, several suggested creating local shelters for long-term care residents or planning for the use of public buildings by facilities that are able to meet residents’ needs in such an environment:

I would really love to see some collaboration between the county office and the healthcare providers to see if we can make this [school] available to you and provide you with generators and security. You do everything else.

#### Conceptual model

Using an abductive approach, we applied the study results to our conceptual model to generate a SEM of disaster preparedness and response in long-term care (Figure 1). This enabled us to examine the multiple levels (e.g., facility/organization, community) at which disaster preparedness and response activities take place in long-term care. Themes and subthemes related to multiple social ecological levels, though uniquely so depending on the level. For example, the subtheme of “collaboration and relationships”, under Theme 1, involved participants’ activities at the facility/organizational level of the model (e.g., reliance on corporate support). Additionally, it involved activities at the larger community level (e.g., relationships with county officials). Figure 1 further illustrates the relationships among the three aspects of disaster preparedness and response reflected in our three themes, with preparatory actions influencing how an event is managed, which leads to disaster effects and consequences that in turn could inform how long-term care facilities anticipate coming disasters.

## Discussion

Florida has ample experience with hurricane preparedness. Florida’s NHs and ALCs are held to state regulations requiring annual county review of disaster plans, but NHs are held to additional federal disaster preparedness regulations (Hyer et al., 2007). Our qualitative interviews and focus groups with NH and ALC administrators identified three major themes: (1) importance of collaborative relationships in anticipating needs and planning to shelter in place or evacuate; (2) efforts required to maintain safety and stability during an unprecedented event; and (3) effects, repercussions, and recommendations for change following the disaster. Subthemes of each indicated specific challenges the administrators faced and their responses. As in prior research (Willoughby et al., 2017), we found evidence of preparedness and response gaps. These gaps, as well as changes prompted by Hurricane Irma, were evident at the level of the facilities themselves (organizational), at a broader community level, and in public policy.

### Providing Safe Spaces in Disasters—From Facility- to Community-Level Factors

Prominent among our results were the factors at multiple levels involved in providing safe physical environments for NH and ALC residents, including whether they evacuate or shelter in place. Evacuation has been found to increase the likelihood of NH resident mortality and hospitalization after a hurricane (Willoughby et al., 2017). The present study provided evidence of residents’ difficult experiences during Hurricane Irma, particularly those who were evacuated.

The decision of whether to leave was made at the facility (organizational) level for most based on their locations, but also on whether they were supplied and equipped to shelter in place. Many participants reported being confident to stay because of their precautions, even some within evacuation zones. However, others chose or were forced by local officials to evacuate, including those not in evacuation zones, because their buildings lacked these protections. Prior research supports these findings (Peterson et al., 2020).

Concerning community-level influences, our results highlight the critical role of local government agencies, primarily emergency managers who issue evacuation orders. Community-level health care coalitions play an important role in these relationships by bringing together health care facilities, emergency managers, and other safety officials, and organizing community disaster drills (Hupert et al., 2015). However, in the present study, only a few participants discussed involvement with these groups.

Much more work is needed to understand obstacles to collaboration between long-term care operators and emergency management officials and to use the findings to develop programs to bring both groups together and communicate the benefits of greater collaboration. Joint discussions should focus on balancing evacuation and sheltering-in-place risks and assessing factors affecting risks, from location and facility maintenance to the availability of alternatives when planned evacuation sites are unavailable. It was not surprising to see in our results that more NHs than ALCs had developed relationships with their emergency managers. Earlier efforts to encourage these relationships focused largely on NHs (Florida Health Care Association, 2007). Future efforts could replicate this work with a focus on ALCs, particularly small ALCs.

Prior research found that ALCs with fewer than 25 beds were more likely than larger ALCs to evacuate for Hurricane Irma (Peterson et al., 2020). Our results suggest that, compared to NHs and larger ALCs, small ALCs are less likely to have corporate support and less likely to have relationships with local emergency managers. Prior research has found that small ALCs are more likely to be independently owned and operated (Caffrey et al., 2014). Castro and colleagues (2008) found after Hurricanes Katrina and Rita in Texas (both in 2005) that small ALCs were more likely than NHs and larger ALCs to evacuate and were less likely to coordinate their responses with local emergency managers. Further research is needed to understand ALC challenges in preparing for and responding to disasters and their need for support from the communities in which they are located. Research also should consider the need to increase ALC regulation or regulatory oversight to improve disaster preparedness or to provide assistance to ALCs that may have difficulty preparing.

A notable finding in this research was that even well-prepared facilities faced challenges (e.g., no-show staff members, generator failure). Many described calling for and receiving assistance from community-level connections in the form of neighbors, residents’ family members, and safety officials patrolling after the hurricane. This highlights the value of communication systems and connections with families. Conversely, some facilities were able to provide help (e.g., sheltering neighbors), highlighting the broader value of NHs and ALCs being part of their communities. Notably, however, a lack of local government assistance left some administrators embittered, convinced they are dependent on private resources in emergencies.

Staffing emerged as a critical concern during Hurricane Irma. Several participants lauded individuals who worked long hours to keep residents safe, providing evidence of the role of interpersonal staff–resident relationships in disaster outcomes and the importance of staff knowing residents’ needs. However, these relationships depend on organizational factors, including staffing practices (e.g., consistent resident assignment), the ability to provide overtime pay, and having administrators who can lead amid the confusion of an emergency.

### The Role of Public Policy in NH and ALC Preparedness and Response

More broadly speaking, however, organizational aspects of staffing cannot be separated from public policies. Disasters increase staffing needs and costs, as suggested in a study of a one-time state of Florida effort to reimburse NHs for 2004–2005 hurricane expenses (Thomas et al., 2010). Recent research found that Hurricane Irma also increased NH staffing costs, particularly for NHs that evacuated, suggesting the need for a renewed effort to help facilities pay for needed disaster staffing (Jester et al., 2021).

The role of public policy was also evident in our results concerning power availability. Many NHs and ALCs reported extended outages after Hurricane Irma, despite pleas to their electric companies. This reflects gaps at an organizational level for facilities lacking generators, and at the community level where power restoration was delayed. However, public policy was also a factor, with the state passing a law after Hurricane Irma requiring NHs and ALCs to have alternate power sources for indoor temperature control. This may improve disaster outcomes by helping to protect NH and ALC residents in a power loss and enabling facilities to shelter in place and more rapidly return to home facilities after evacuation. We note, however, that extended power losses after Hurricane Irma strained generators’ capabilities. Further policy efforts are needed to include NHs and ALCs among the providers receiving priority for power restoration after a disaster.

Public policy is a factor in every aspect of disaster preparedness and response, due to government preparedness requirements. However, we found variation in participants’ preparations, from those who simply completed required forms to those with systems to assess their needs and capabilities and prepare accordingly. These finding show adequate preparedness is more complex than simply establishing regulations, echoing prior research (Dosa et al., 2008) and adding to findings that federal NH rules fall short of generating community connections often needed in a disaster (Lane & McGrady, 2018). Hurricane Irma did offer lessons to Florida’s NHs and ALCs. Several participants discussed organizational changes to improve their responses in the future. However, more work is needed. Organizational and policy changes resulting from Hurricane Irma may not be enough without improved community-level connections, particularly with health care coalitions and emergency managers.

### Limitations

While this study is one of the first to broadly examine experiences of NHs and ALCs related to disaster preparedness, the study has limitations. As a qualitative study, results cannot be generalized to the larger long-term care facility population. Additionally, participants may have had difficulty recalling events, given the time between the storm and our interviews. Some participants had moved to different facilities. We attempted to mitigate these challenges by prioritizing responses from those who had not changed positions and asking participants to refer to reports they were required to create at the time of the hurricane. Further, some participants represented multiple locations. We prompted them to be specific whenever possible. Data collection was limited to those in administrative positions, given their involvement in the multiple aspects of writing and executing a disaster plan and in managing a disaster. Future research including emergency managers, care staff, residents, family members, and other community members would strengthen the results of the present research.

## Conclusion

Our results highlight the importance of long-term care organizations building connections with stakeholders, emergency managers, community organizations, and others who can provide support in a disaster. These lessons also apply to the challenges of managing the COVID-19 pandemic in NHs and ALCs. Both natural disasters and pandemics create disruptions at organizational, community, and societal levels. Policy changes have the potential to improve NHs’ and ALCs’ disaster preparedness. Mandating the vital relationships may be difficult, but more work involving emergency managers is needed to reduce the perception that NHs and ALCs are on their own during disasters.

## Supplementary Material

igab038_suppl_Supplementary_Materials
