# Supplementary material for: “You Just Forge Ahead”: The Continuing Challenges of Disaster Preparedness and Response in Long-Term Care
Source: Innov Aging. 2021 Sep 18;5(4):igab038. doi: 10.1093/geroni/igab038 (PMC8600546; doi:10.1093/geroni/igab038)
Supplement: igab038_suppl_Supplementary_Materials [file igab038_suppl_supplementary_materials.docx]

Supplementary Table 1. Characteristics of Participants (N=89)

| Characteristic | Total (n/%) |
| --- | --- |
| Female | 53 (59.6) |
| Race |  |
| White | 80 (89.9) |
| Black | 8 (9.0) |
| Asian | 1 (1.1) |
| Ethnicity |  |
| Non-Hispanic | 73 (82.0) |
| Hispanic | 16 (38.0) |
| Position |  |
| Administrator/Manager | 48 (53.9) |
| Owner | 19 (21.3) |
| Executive Director/CEO/VP | 12 (13.5) |
| Director of Nursing/Assisted Living | 5 (5.6) |
| Other | 5 (5.6) |
| Long-Term Care Experience |  |
| Less than 6 years | 14 (15.7) |
| 6-20 years | 39 (43.8) |
| 21 or more years | 26 (29.2) |
| Did not say | 10 (11.2) |
| Disaster Experience |  |
| Hurricanes Only | 39 (43.8) |
| Other Disasters Only | 3 (3.4) |
| Hurricanes and Other Disasters | 16 (18.0) |
| None | 18 (20.2) |
| Did not say | 13 (14.6) |

*Note*. Five participants discussed more than one nursing home or assisted living community.

Supplementary Table 2. Nursing Home and Assisted Living Community Characteristics (N=99)

| Characteristics | Small ALC (<25 beds) (n=32) | Large ALC (25+ beds) (n=38) | NH  (n=29) |
| --- | --- | --- | --- |
| Evacuated (n/%) | 15 (46.9) | 15 (39.5) | 14 (48.3) |
| Total Beds (M/SD) | 9.4 (4.4) | 112.6 (56.6) | 127.9 (62.1) |
| For-profit (n/%) | 32 (100) | 28 (73.7) | 15 (51.7) |
| License Type (n/%) |  |  |  |
| Standard Only | 18 (56.3) | 13 (34.2) | NA |
| ECC and/or LNS | 6 (18.8) | 23 (60.5) | NA |
| LMH | 8 (25.0) | 2 (5.3) | NA |
| Memory Care (n/%) | 5 (15.6) | 12 (31.6) | NA |
| Accepts OSS Payment (n/%) | 13 (40.6) | 6 (15.8) | NA |
| Accepts Medicaid (n/%) | 14 (43.8) | 20 (52.6) | NA |
| Chain Membership (n/%) | 13 (40.6) | 22 (57.9) | 21 (72.4) |
| Building Type (n/%) |  |  |  |
| Freestanding | 31 (96.9) | 16 (42.1) | 21 (72.4) |
| ALC/NH | 0 (0) | 9 (24.3) | 0 (0) |
| IL/ALC | 0 (0) | 2 (5.4) | NA |
| IL/ALC/NH | 1 (3.1) | 11 (29.7) | 8 (31.8) |
| Have Alzheimer’s Disease Beds (n/%) | NA | NA | 10 (34.5) |
| Occupancy Rate (M/SD) | NA | NA | 87.9 (8.1) |
| Medicaid Occupancy (M/SD) | NA | NA | 52.1 (26.3) |

*Note*. NA=Not Applicable or Not Available, ECC = Extended Congregate Care, LNS = Limited Nursing Services, LMH = Limited Mental Health, ALC = Assisted Living Community, NH = Nursing Home, IL = Independent Living, OSS = Optional State Supplementation. One evacuated nursing home did not have data available from CMS.

Supplementary Data 1. Interview and Focus Group Guide, Hurricane Irma

*We are with the University of South Florida School of Aging Studies. We are gathering information to understand the experiences of assisted living and nursing home administrators during Hurricane Irma. We are conducting interviews and focus groups with administrators across the state whose facilities were affected by Hurricane Irma. We are interested in all your thoughts and suggestions; there are no right or wrong answers. Everything you say is confidential and will not be shared with anyone other than the researchers on this project.*

Can I answer any questions for you before we begin?

1. Can you tell us about yourself and your experience with other disasters (type of disaster?) Length of time in current job/in industry?
2. Can you tell us about your evacuation/shelter in place experience during Hurricane Irma?
   1. Prompts- How did families communicate with you? How did you communicate with residents’ families about the need to evacuate/shelter in place and their options to care for residents during the hurricane? What role did family communication or residents’ ability to self-evacuate have on the decision to evacuate/shelter in place?
   2. If you evacuated, did you evacuate before or after the hurricane?
3. Can you characterize the residents who went home with family versus those that evacuated with you?
4. Who made the ultimate decision to evacuate your facility or to shelter in place?
   1. Prompt if needed - Was it completely yours, completely another (who?), corporate? Driven by orders from emergency management center?
   2. How did the media and weather channel reporting influence your decisions?
5. Please respond to the following statement on a 1- 5 scale with 1 (strongly agree to -5 strongly disagree “I felt pressure from others in making this decision?” Prompt - Please expand on any pressure you may have felt and the source of that pressure.
6. Please respond to the following statement on a 1- 5 scale with 1 (strongly agree to -5 strongly disagree), I felt I made an informed choice.
   1. What other information did you need?
   2. What information was critical to your decision?
7. About how many hours did it take to evacuate from your facility? ( Differentiate preparation from travel time to destination(s))
8. Were any residents from your community injured during the hurricane

Can you discuss how the experience affected residents with dementia? Can you elaborate? What might be done differently in future storms?

1. If you evacuated, please tell us about the place(s) (facility) you evacuated to and how you/they met the residents’ special needs (e.g. with stretchers, oxygen, dialysis).
   1. Provide some information on where residents were housed and if it varied by resident condition (e.g. dining room, individual beds)
   2. Was this place part of your comprehensive emergency management plan and mutual agreement?
2. On a scale from 1-5, with 1 being minimal collaboration and 5 being a high level of collaboration, how would you rate your level of collaboration with emergency operations center (EOC) or state agencies in the evacuation or the sheltering in place of your residents?
   1. Please elaborate on any EOC collaboration issues you may have had.
3. On same scale, from 1-5, with 1 being minimal collaboration and 5 being a high level of collaboration, how would you rate the transportation available to you, your residents in this evacuation?
   1. Please elaborate on transportation collaboration issues you may have had.
4. Can you describe your experiences with water and power restoration?
5. Can you tell us about returning to the NH/AL?
   1. Prompt- When did your residents return to your NH/AL?
   2. How did your residents adjust upon their return to your assisted living after the evacuation?
   3. When did the families return residents to the NH/AL? Did all residents who evacuated with family return?
6. Please response to the following statement on a 1- 5 scale with 1 (strongly agree to -5 strongly disagree), “I am satisfied with my decision to evacuate or to shelter in place.”
   1. Prompt - Can you elaborate?
7. Overall, please rate your level of preparedness for Hurricane Irma (Please answer from 1-5, with one indicating a low level of preparedness and 5 indicating a high level).
   1. Prompt - Can you elaborate?
8. Since the hurricane, have you revised or discussed revising your disaster plan?
   1. Prompt - What areas, types of things do you anticipate changing? What concerns are beyond your control? Why?
9. How many of your residents are on Medicare Advantage instead of Fee-for-Service?
10. Is there anything else you would like to add or emphasize

*Thank you for agreeing to this interview. You have helped us understand a complex and important issue.*
